# Supplementary material for: Fasting Proinsulin Independently Predicts Incident Type 2 Diabetes in the General Population
Source: J Pers Med. 2022 Jul 12;12(7):1131. doi: 10.3390/jpm12071131 (PMC9323856; doi:10.3390/jpm12071131)
Supplement: Supplementary file 1 [file jpm-12-01131-s001.zip › jpm-1785645-supplementary.pdf]

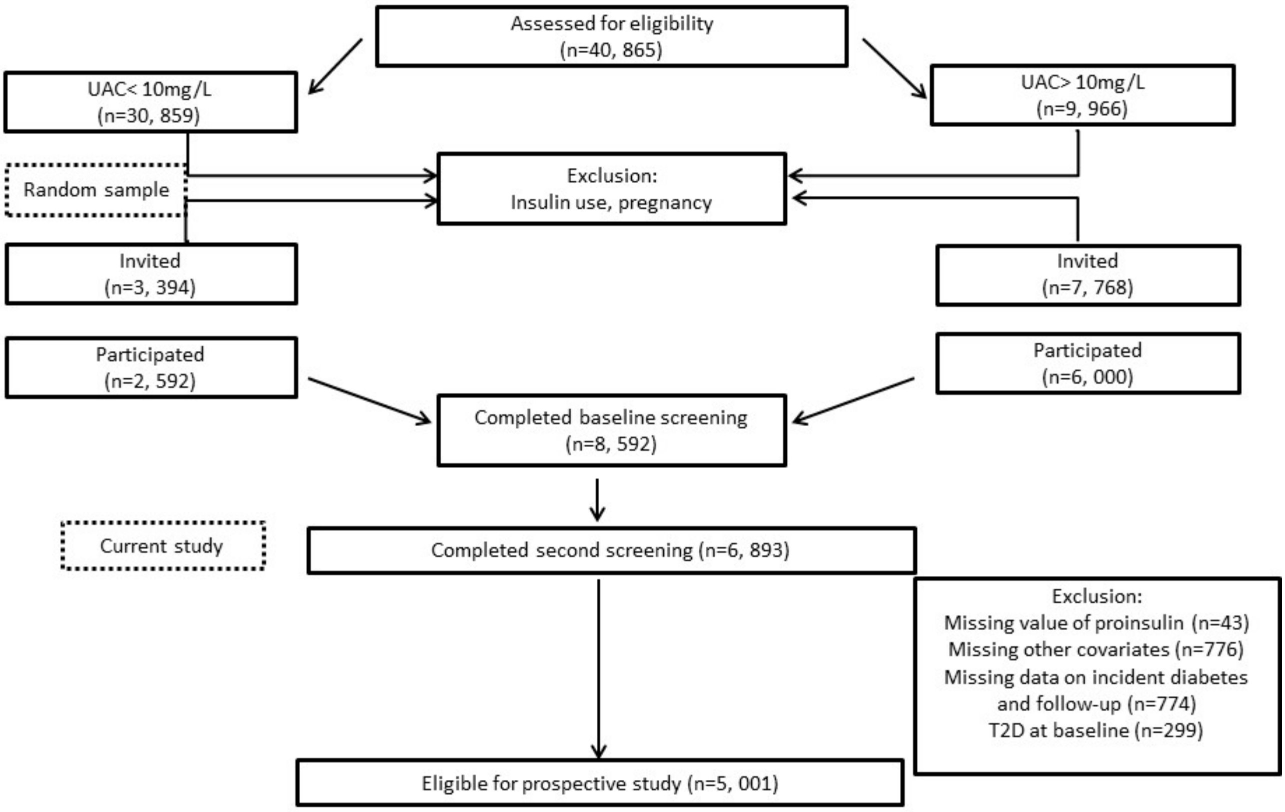

Figure S1. Detailed information on reason of exclusion and numbers of missing values of proinsulin measurement ,other covariates, and loss to follow-up for the analyses on proinsulin and their association with incident type 2 diabetes (T2D) in The PREVEND study.

Table S1. Uni and Multi variable adjusted linear regression analyses with log-transformed proinsulin as dependent variable in 5,001 participants of the PREVEND study.

Variables	Univariable		Multivariable adjusted (R^2=0.442)	
	Standardized ß	*p* value	Standardized ß	*P* value
Sex, female vs. male	-0.214	<0.001	-0.125	<0.001
Age, years	0.294	<0.001	0.072	<0.001
Family history of Diabetes, yes vs. no	0.073	<0.001	0.014	0.222
Current smoking, yes vs. no	-0.014	0.33	-	-
Alcohol, yes vs. no	-0.065	<0.001	-0.007	0.552
BMI, kg/m²	0.432	<0.001	0.076	<0.001
Systolic blood pressure, mm Hg	0.307	<0.001	0.008	0.657
Antihypertensive medication, yes vs. no	0.219	<0.001	-0.009	0.573
Cholesterol, mmol/L	0.110	<0.001	-0.032	0.021
HDL cholesterol, mmol/L	-0.306	<0.001	0.002	0.902
Triglycerides, mmol/L	0.372	<0.001	0.064	<0.001
Lipid-lowering medication, yes vs. no	0.158	<0.001	0.017	0.143
Glucose, mmol/L	0.379	<0.001	0.112	<0.001
Insulin (mU/L)	0.537	<0.001	0.127	<0.001
C-peptide (pmol/L)	0.613	<0.001	0.333	<0.001
eGFR, mL/min/1.73 m^2	-0.268	<0.001	-0.019	0.197
Urinary albumin excretion, mg/24 hours	0.243	<0.001	0.054	<0.001

BMI: body mass index; HDL: high-density lipoprotein; eGFR: estimated glomerular filtration rate; PREVEND: Prevention of renal and vascular End-stage Disease

Table S2.Competing risk model of proinsulin to mortality and incident T2D in 5001 participants without diabetes.

	Subhazard Ratio (95% CI)	*p* value
Crude analysis	2.57 (2.25–2.93)	<0.001
Model1	2.47 (2.16–2.83)	<0.001
Model2	2.45 (2.14–2.80)	<0.001
Model3	1.98 (1.71–2.31)	<0.001
Model4	1.82 (1.57–2.11)	<0.001
Model5	1.85 (1.59–2.15)	<0.001
Model 6	1.32 (1.11-1.57	0.002
Model 7	1.27 (1.08-1.59)	0.005
Model 8	1.23 (1.00–1.51)	<0.044

Subhazard Ratio (95% CIs) were derived from competing risk analyses by Fine and Gray .
Model 1 is adjusted for age and sex.
Model 2 is additionally adjusted for smoking status and alcohol consumption.
Model 3 is additionally adjusted for BMI, family history of diabetes and hypertension.
Model 4 is additionally adjusted for triglycerides, total cholesterol and HDL cholesterol.
Model 5 is additionally adjusted for eGFR and urinary albumin excretion.
Model 6 is additionally adjusted for glucose
Model 7 is additionally adjusted for insulin
Model 8 is additionally adjusted for C-peptide
eGFR: estimated glomerular filtration rate; PREVEND: Prevention of renal and vascular End-stage Disease.
